# Crystallization and initial X-ray diffraction analysis of the multi-domain *Brucella* blue light-activated histidine kinase LOV-HK in its illuminated state

**DOI:** 10.1016/j.bbrep.2018.09.005

**Published:** 2018-09-26

**Authors:** Jimena Rinaldi, Ignacio Fernández, Lucía M. Poth, William E. Shepard, Martin Savko, Fernando A. Goldbaum, Sebastián Klinke

**Affiliations:** a Fundación Instituto Leloir, IIBBA-CONICET, Av. Patricias Argentinas 435, Buenos Aires C1405BWE, Argentina; b Unité de Virologie Structurale, Département de Virologie, Institut Pasteur, 25 Rue du Dr. Roux, Paris 75015, France; c Synchrotron SOLEIL, L′Orme des Merisiers, Saint-Aubin BP 48, Gif-sur-Yvette Cedex, 91192, France; d Plataforma Argentina de Biología Estructural y Metabolómica PLABEM, Av. Patricias Argentinas 435, Buenos Aires C1405BWE, Argentina

**Keywords:** Multi-domain protein, Light activation, Signal transduction, Two-component system, Histidine kinase

## Abstract

The pathogenic bacterium *Brucella abortus* codes for a multi-domain dimeric cytoplasmic histidine kinase called LOV-HK, which is a key blue light-activated virulence factor in this microorganism. The structural basis of the light activation mechanism of this protein remains unclear. In this work, full-length LOV-HK was cloned, expressed and purified. The protein was activated by light and crystallized under a controlled illumination environment. The merge of 14 individual native data sets collected on a single crystal resulted in a complete X-ray diffraction data set to a resolution of 3.70 Å with over 2 million reflections. Crystals belong to space group *P*2_1_2_1_2_1_, with unit-cell parameters a = 95.96, b = 105.30, c = 164.49 Å with a dimer in the asymmetric unit. Molecular replacement with Phaser using the individual domains as search models allowed for the reconstruction of almost the whole protein. Very recently, improved LOV-HK crystals led to a 3.25-Å resolution dataset. Refinement and model building is underway. This crystal model will represent one of the very few examples of a multi-domain histidine kinase with known structure.

## Highlights

• The LOV-HK histidine kinase from *Brucella* was crystallized in its activated state.
• A strict illumination control was followed for protein crystallization.
• Highly redundant X-ray diffraction data were collected at 3.70 Å maximum resolution.
• Phasing was achieved in a tricky molecular replacement case.

## Graphical abstract

**Figure:**
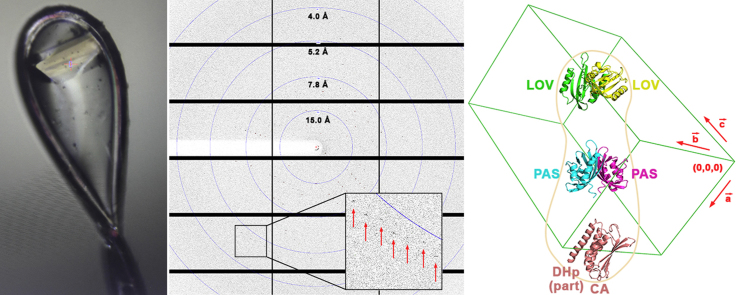

## 1 Introduction

*Brucella abortus* is a Gram-negative intracellular bacterium that affects cattle causing brucellosis, a worldwide disease that can be transmitted to humans. It has been shown that exposure of *B. abortus* to visible light results in a 10-fold higher level of bacterial replication in mouse macrophages than the corresponding dark control [1]. This light-dependent virulence enhancement is mediated by a cytoplasmic sensor histidine kinase called LOV-HK, which is part of a two-component signal transduction system involved in the modulation of the general stress response in *Brucella* [2].

**Fig. 1:**
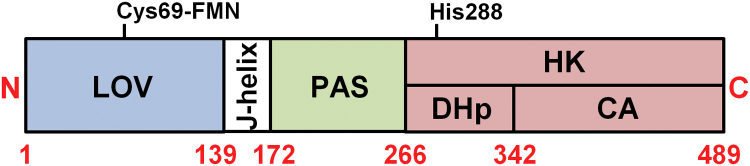
Schematic representation of the domain organization in LOV-HK. The relevant residues Cys69 and His288 are also marked in their approximate locations (see the text for details).

LOV-HK is a 489-residue, 108-kDa dimeric protein formed by three domains, namely LOV, PAS and HK (Fig. 1). LOV (Light-Oxygen-Voltage, residues 1–139) corresponds to the blue-light sensor domain through a bound FMN cofactor. Upon light absorption, a covalent bond is generated between the S^γ^ atom of the Cys69 residue and the C4(a) atom of the ligand, which disrupts part of the aromatic structure of the latter molecule starting the signal transduction cascade by a yet unknown mechanism [3]. This domain is followed by PAS (Per-Arnt-Sim, 172–266), with unknown function, and HK (Histidine Kinase, residues 267–489), which autophosphorylates upon light absorption at a conserved histidine residue (His288) through a bound ATP molecule [4]. The latter domain is divided in two subdomains: (i) the first called DHp (Dimerization and Histidine phosphotransfer, 267–342), which is a helical hairpin linker that generates a four-helix bundle in the protein dimer, and (ii) the second called CA (Catalytic and ATP binding, 343–489), which is a globular compact domain whose relative orientation with respect to the rest of the molecule is dependent on the particular functional state of the kinase, as proposed for the *Bacillus subtilis* DesK sensor HK [5]. The LOV and PAS domains are linked by a long predicted helical element called the J-helix, and together with the DHp subdomain, these α-helices are hypothesized to be key actors in the downstream signal transduction events from the LOV domain to the HK domain by means of a series of structural rearrangements.

Interestingly, there are just five two-domain and one three-domain HKs with known crystal structures. The latter protein corresponds to the cytoplasmic portion of VicK from *Streptococcus mutans*, which bears the HAMP-PAS-HK domain triad (PDB 4I5S) [6]. In all cases, the sequence identity of these proteins in comparison with LOV-HK is below 25%.

Over the past few years, we have been able to solve the crystal structures of the individual domains of LOV-HK, namely the LOV domain in the dark (PDB code 3T50) [3], the PAS domain (from a LOV-PAS structure in the dark, Rinaldi *et al.*, unpublished results), and the HK domain (PDB code 5EPV) [4], [7]. However, there is a lack of structural information for the protein as a whole and the changes that are triggered by light. Here, we present the crystallization and initial X-ray data analysis of the illuminated form of LOV-HK and describe its phasing by molecular replacement using the available domain fragments as search models. This model will greatly complement the existing knowledge of this complex system and will provide essential information regarding the activation of sensor HKs in general.

## 2 Materials and methods

### 2.1 Macromolecule production

**Fig. 2:**
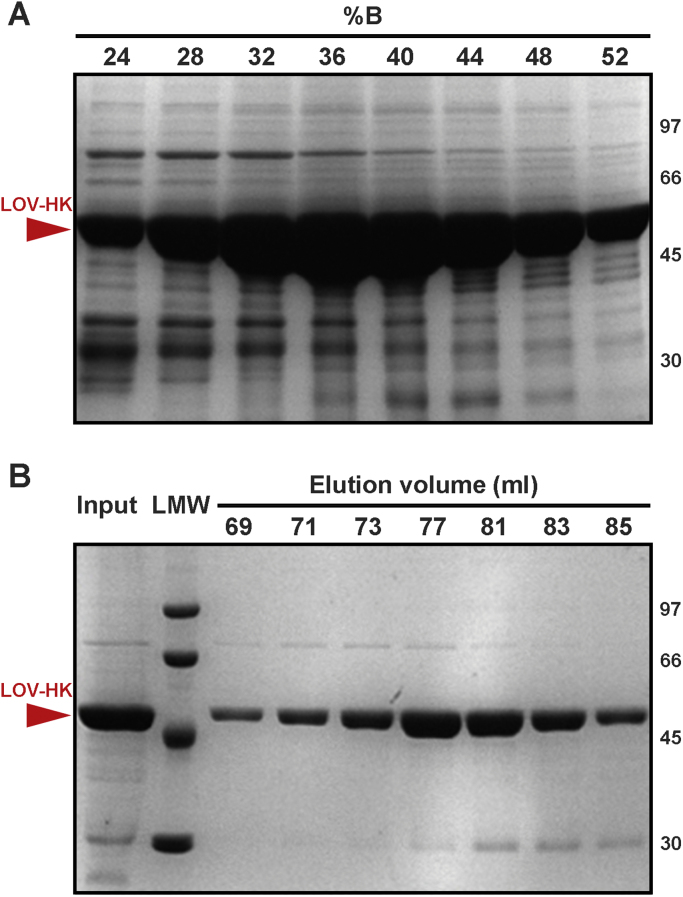
12% Coomassie Blue-stained SDS–PAGE gels of the purification steps. The MWs corresponding to standard markers (LMW, kDa) are indicated at the right. (A) His-trap step, with selected fractions 32–52% buffer B. (B) Superdex 200 step, with selected fractions 69–85 ml.

The gene coding for LOV-HK (UniProt accession code Q2YKK7) was produced by restriction-free cloning with the oligonucleotide primers indicated in Supplementary Table S1. Briefly, a first PCR was run using the primers and *B. abortus* genomic DNA as template, and the obtained fragment served as megaprimer in a second PCR with the pET-24a cloning vector as template. *Dpn*I was used to degrade the template DNA. The quality of the obtained construct, named pET-24a-LOVHK-15–489, was assessed by DNA sequencing. It includes a single N-terminal residue cloning artifact (Met) followed by the coding region for almost the complete protein (15−489) with the exception of its first 14 residues, which are predicted to be disordered by the DISOPRED3 server [8]. The construct bears a 6x His tag at its C-terminus, giving rise to a total of 482 residues (Supplementary Table S1). *Escherichia coli* BL21(DE3)pLysS cells were transformed with the pET-24a-LOVHK-15–489 construct and grown overnight in 5 ml of LB medium added with 35 µg ml^−1^ kanamycin at 37 °C with agitation (250 rev min^−1^). It is important to note that all the following steps of protein production and purification were performed in the dark, either in special adapted rooms or using laboratory glass material and other equipment covered with aluminum foil. Precultures were diluted in 500 ml of ZYM-5052 auto-inducing medium [9] and grown initially for 3 h at 37 °C and then overnight at 28 °C with agitation (200 rev min^−1^). Bacteria were centrifuged at 10,000*g* for 8 min at 4 °C. The pellet was resuspended and sonicated in a solution consisting of 50 mM Tris, 0.5 M sodium chloride, 20 mM imidazole, 1 mM PMSF, 1 mM DTT, pH 8.2 (buffer A) and then centrifuged at 160,000*g* in a Beckman Coulter L7–65 ultracentrifuge (Brea, California, USA) for 1 h at 4 °C. The supernatant was filtered through a 0.45 µm membrane and loaded onto a HisTrap HP column (all columns were from GE Healthcare, Little Chalfont, England) in a Gilson FPLC apparatus (Luton, England). Elution was performed with a linear gradient of buffer B consisting of 50 mM Tris, 0.5 M sodium chloride, 0.5 M imidazole, 1 mM PMSF, 1 mM DTT, pH 8.2. A major peak was observed at around 40% buffer B (Fig. 2A and Supplementary Fig. S1A). The appropriate protein fractions were pooled and dialyzed overnight at 4 °C against buffer C (50 mM Tris, 0.25 M sodium chloride, 1 mM PMSF, 0.5 mM DTT, pH 8.2) and further purified by gel filtration chromatography on a Superdex 200 16/60 column with isocratic elution in buffer C. A major peak was observed at around 78 ml (Fig. 2B and Supplementary Fig. S1B). The final protein fractions were then concentrated to approximately 7 mg ml^−1^ by centrifugation in Amicon Ultra-4 devices (Millipore, Billerica, Massachusetts, USA) and simultaneously exchanged into lower ionic strength crystallization buffer (10 mM Tris, 100 mM sodium chloride, pH 8.2). The concentration of the sample was estimated by using the calculated molar extinction coefficient at λ = 280 nm provided by the ExPASy ProtParam tool based on the polypeptide sequence (ε = 95,800 M^−1^ cm^−1^) [10], subtracting approximately 25% of the total absorbance coming from the contribution of the FMN cofactor in the dark. For this purpose, an absorbance standard calibration curve of this ligand was used. The protein was aliquoted, flash frozen in liquid nitrogen and stored at −70 °C. The quality of the final preparation was assessed by SDS–PAGE (Fig. 2), UV–Vis spectrophotometry (Supplementary Fig. S2) and static light scattering (Supplementary Fig. S3).

### 2.2 Crystallization

**Fig. 3:**
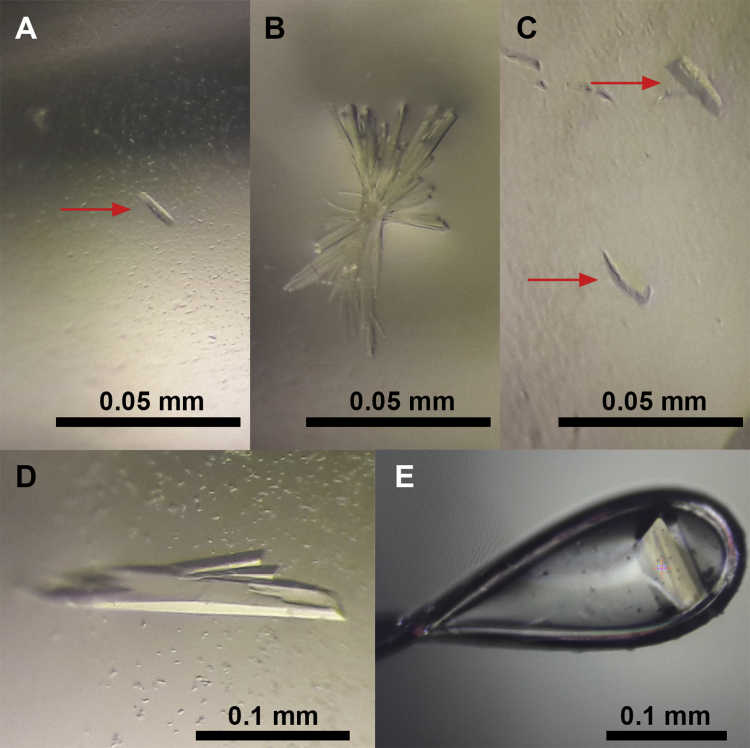
LOV-HK crystals. (A) Screen hit #1, obtained with 30% (*v/v*) MPD, 5% (*w/v*) PEG 4000, 0.1 M Hepes, pH 7.5 (Jena Bioscience JBScreen Classic 7 Solution C4). (B) Screen hit #2, obtained with 5% (*v/v*) isopropanol, 0.1 M Hepes, pH 7.5 (Jena Bioscience JBScreen Classic 9 Solution A1). (C) Screen hit #3, obtained with 10% (*w/v*) PEG 4000, 20% (*v/v*) isopropanol, 0.1 M Hepes, pH 7.5 (Jena Bioscience JBScreen Classic 3 Solution A5). (D) Improved crystals obtained after optimization of screen hit #1. (E) Mounted crystal at the PROXIMA-2A beamline, ready for diffraction.

The protein samples were thawed in the dark in ice and then subjected to a 10 min white light illumination pulse at room temperature (10 µmol m^−2^ s^−1^), with the addition of 3 mM magnesium chloride and 3 mM AMP-PCP (a non-hydrolyzable ATP analogue). Next, the protein was centrifuged at 21,000*g* for 10 min at 10 °C to remove any precipitate generated at the activation step. Initial crystallization trials were performed at 5.3 mg ml^−1^ in 96-well sitting-drop vapour-diffusion Greiner 609120 plates (Monroe, North Carolina, USA) using a Honeybee 963 robot (Digilab, Marlborough, Massachusetts, USA) and the following crystallization kits: Jena Bioscience JBScreen Classic and Pentaerythritol (Jena, Germany), and Hampton Research Crystal Screen, Crystal Screen 2, PEG/Ion, PEG/Ion 2, PEGRx 1 and PEGRx 2 (Aliso Viejo, California, USA). Plates were stored at 21 °C under white light pulsed illumination (40 µmol m^−2^ s^−1^, 1 min every 6 h). After several days of equilibration, three out of the 576 conditions tested revealed promising crystal hits consisting of tiny imperfect yellowish bars (screen hits #1, #2 and #3, Fig. 3A-C). These three conditions were optimized manually with success, and the best diffracting crystals were obtained after improvement of the conditions that yielded screen hit #1, namely 3.5–10.0% (*w/v*) PEG 4000, 15–30% (*v/v*) MPD, 0.1 M Hepes, pH 7.2–7.8, and under the same illumination protocol as for the robotic screen (Fig. 3D). Samples were cryoprotected in mother liquor with a higher MPD concentration following the empirical rule % _MPD_ + % _PEG 4000_ = 35 (which proved to be successful for all crystals tested) and then cooled in liquid nitrogen in Hampton Research loops (Fig. 3E).

### 2.3 Data collection and processing

**Fig. 4:**
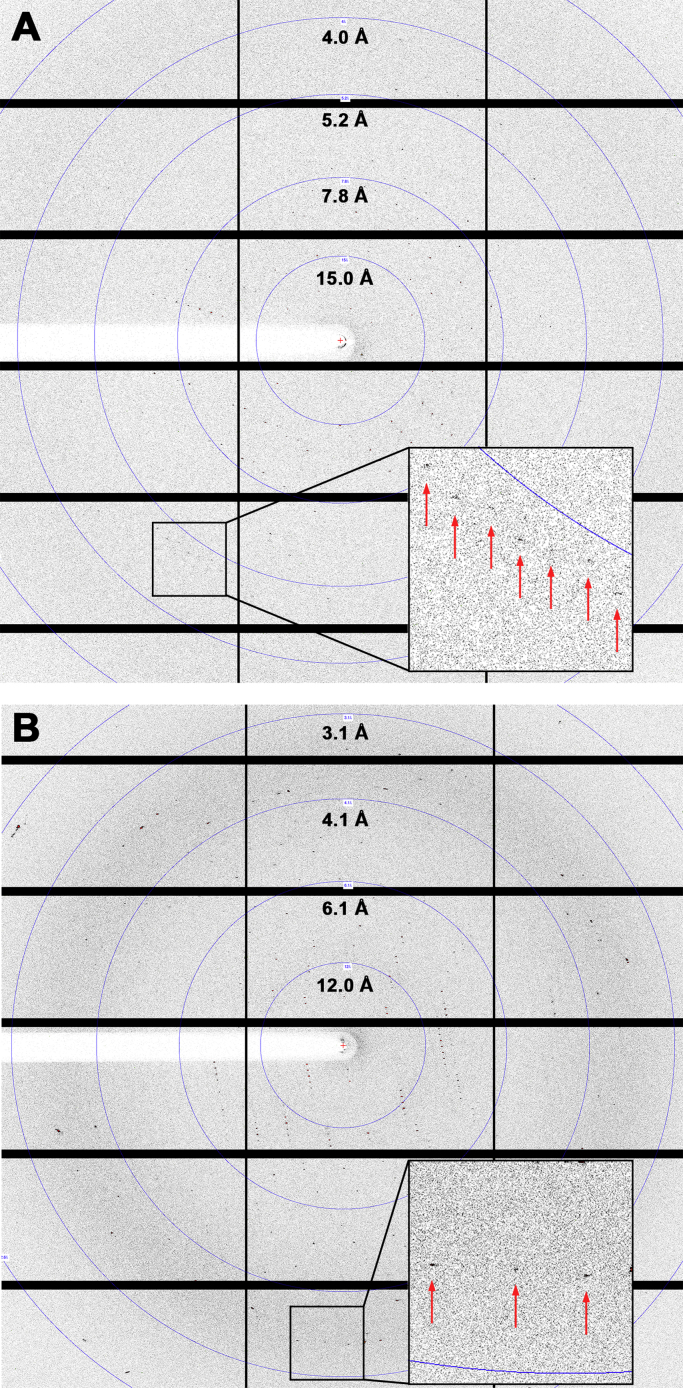
LOV-HK diffraction patterns as shown with ALBULA (Dectris, Baden, Switzerland). Resolution rings are depicted, and magnified regions with the spot locations indicated by arrows are presented for better visualization. (A) Crystal #1. (B) Crystal #2.

Single crystal X-ray diffraction data were measured at the PROXIMA-2A microfocus protein crystallography beamline at Synchrotron SOLEIL (France) on a few dozen crystals. Fig. 4A shows a diffraction pattern corresponding to the best crystal from the initial batch (Crystal #1, Table 1, grown with 4.5% (*w/v*) PEG 4000, 22% (*v/v*) MPD, 0.1 M Hepes, pH 7.5). Depending on the diffraction quality observed and the particular crystal shape, both standard and helical data collection protocols [11] were followed using the MXCuBE application [12]. X-ray diffraction data were processed with XDS [13] using the *xdsme* command-line interface (https://github.com/legrandp/xdsme) and scaled using Aimless [14]. For cross validation purposes, 5% of the recorded reflections were flagged apart. Complete information on data collection parameters and processing statistics is presented in Table 1.

**Table 1:** X-ray data collection and processing.

	Crystal #1	Crystal #2
Diffraction source	PROXIMA-2A, SOLEIL	PROXIMA-2A, SOLEIL
Wavelength (Å)	0.9801	0.9801
Temperature (K)	100	100
Detector	EIGER X 9 M	EIGER X 9 M
Number of individual datasets	14	1
Crystal-detector distance (mm)	391.87–410.27	317.67
Rotation range per image (°)	0.1	0.1
Total rotation range (°)	180–360	400
Exposure time per image (s)	0.025–0.100	0.025
Space group	*P*2_1_2_1_2_1_	*P*2_1_2_1_2_1_
a, b, c (Å)	95.96, 105.30, 164.49	95.96, 104.66, 164.83
α, β, γ (°)	90, 90, 90	90, 90, 90
Mosaicity (°)	0.092–0.148	0.120
Resolution range (Å)	64.82–3.70	62.53–3.25
Total No. of reflections	2,071,362	396,552
No. of unique reflections	18,388	26,861
Completeness (%)	100.0 (100.0)	100.0 (100.0)
Redundancy	112.6 (107.2)	14.8 (15.3)
〈I/σ(I)〉	24.2 (1.1)	13.1 (1.0)
*R*_meas_	0.186 (4.117)	0.118 (2.774)
CC_1/2_ (%)	100.0 (72.0)	99.8 (60.9)
Overall *B* factor from Wilson plot (Å^2^)	196	92

Values for the outer shell are given in parentheses: Crystal #1, 3.80–3.70 Å; Crystal #2, 3.47–3.25 Å.

## 3 Results and discussion

LOV-HK could be successfully expressed, with an approximate yield of 9 mg per liter of bacterial culture at the end of the purification process. The SDS-PAGE gel run after the affinity chromatography step (Fig. 2A) revealed a major LOV-HK band whose MW is in good agreement with the 54 kDa value calculated from the polypeptide sequence, and there is over 95% purity in the final preparation, with two very weak contamination bands noticeable after the size exclusion chromatography step (Fig. 2B). Furthermore, a static light scattering (SLS) analysis coupled to size exclusion chromatography validated that LOV-HK exists as a dimer in solution with an experimental MW of 110 ± 3 kDa (Supplementary Fig. S3).

With respect to the robotic crystallization screen, it is interesting to note that all solutions that yielded preliminary crystals were buffered with 0.1 M Hepes at pH 7.5 (Fig. 3A-C), which is not far from the expected pI of the protein (6.9). Optimization of the crystallization conditions gave rise to bigger crystals, with a maximum size of 0.3 mm x 0.1 mm x 0.1 mm, and always with a central longitudinal groove as can be appreciated in Fig. 3D and E.

In general, the diffraction quality of the illuminated LOV-HK crystals and hence the maximum resolution reached was well in line with their size. The best crystal from our first batch of samples (Crystal #1, June 2017) diffracted X-rays initially to 3.90 – 4.20 Å in different locations (Fig. 4A), with a quality that remained relatively constant along the crystal. For this reason, we decided to collect a series of complete individual datasets applying both standard as well as helical data collection protocols, in order to improve the statistics and reach a better resolution. A total of 14 datasets collected in different parts of the crystal were merged (3 standard + 11 helical) and a maximum resolution of 3.70 Å was achieved with over 2 million individual spots, as described in Table 1.

**Fig. 5:**
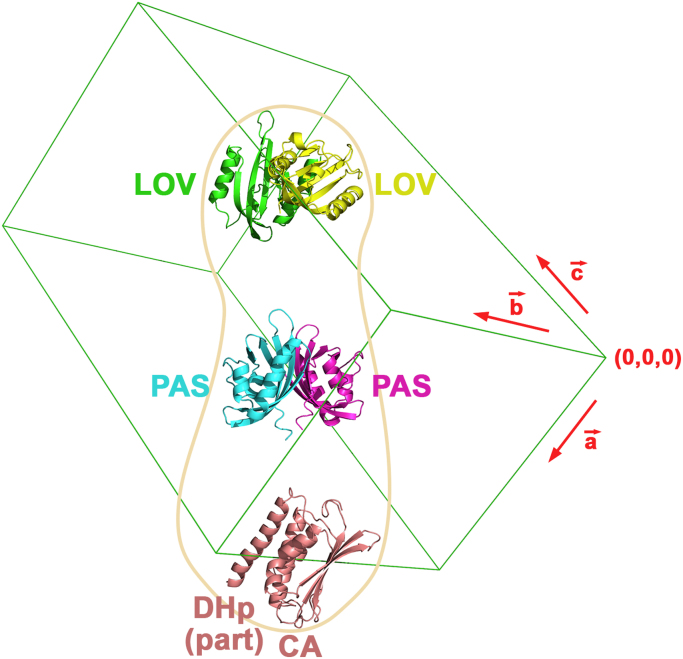
Domains successfully located by molecular replacement with Phaser. All depicted domains belong to the same LOV-HK dimer, and the approximate shape expected for the complete molecule is contoured in light brown. The unit cell is shown in green. The figure was drawn with PyMOL (Schrödinger, New York, USA).

An analysis on the solvent content [15] indicated 40% probability of having a single dimer in the asymmetric unit (*V*_M_ = 3.83 Å^3^ Da^−1^ and 68% solvent), and 58% probability of having two dimers (*V*_M_ = 1.91 Å^3^ Da^−1^ and 36% solvent). These two possibilities, together with the expected inter-domain flexibility commonly observed in histidine kinases in particular and in multi-domain structures in general, and the low resolution of the available diffraction data, made this particular case a challenging example for molecular replacement despite the existing X-ray structures of the individual domains of the protein. In this sense, initial attempts were performed with Phaser [16] as implemented in the CCP4 suite [17], using the following search models: LOV (residues 21–135), PAS (172−273) and HK (311–479, partial domain including part of the DHp and the complete CA subdomain). Different combinations of multi-ensemble searches in a trial-and-error manner were sequentially carried out. This lead to the successful location of five domains, namely two LOV, two PAS, and one copy of the HK fragment mentioned above, all belonging to the unique dimer eventually present in the asymmetric unit (Fig. 5). The statistics obtained after this step were *Translation Function Z-score* = 18.8, *R* = 0.566 and *Refined LLG* = 1274. Although the values for the *Translation Function Z-score* and the *Refined LLG* are representative of a correctly solved structure, the high *R* factor obtained at this step may be the consequence of the low resolution of the diffraction data, the high solvent percentage and the lack of one of the globular domains of the protein and all bridging elements in the model in the current stage. It is interesting to note that all molecular replacement attempts performed using LOV-LOV, PAS-PAS and HK-HK dimers as search models did not yield favorable results, probably due to internal rearrangements in the complete protein.

Initial model building and refinement cycles using Coot [18] and Refmac [19], respectively, allowed for the preliminary placement of most of the helical connecting elements bridging the LOV, PAS and HK domains. In parallel, we were able to improve the size and diffraction quality of the crystals, reaching 3.25 Å resolution with a new batch of samples (Crystal #2, Table 1 and Fig. 4B, November 2017, grown with 4.0% (*w/v*) PEG 4000, 15% (*v/v*) MPD, 0.1 M Hepes, pH 7.2). At the moment, we are actively working in model building and refinement of the structure using these new data.

It is important to mention that we were also able to grow LOV-HK crystals in the dark following a strict light control protocol and in the same condition that yielded Screen hit #2 for the illuminated protein (5% (*v/v*) isopropanol, 0.1 M Hepes, pH 7.5, Jena Bioscience JBScreen Classic 9 Solution A1), but unfortunately the best diffraction signal obtained for these samples reached only 6 Å resolution. In conclusion, taking into account that the structures of the LOV and LOV-PAS constructs in the dark have already been solved in our lab, the structure of LOV-HK in its illuminated state will provide invaluable information regarding the light-driven activation of the protein and its inter-domain rearrangements upon light absorption, and may reveal further details of the molecular mechanism and plasticity that govern activation in sensor HKs.
