# Supplementary material for: Crystallization and initial X-ray diffraction analysis of the multi-domain Brucella blue light-activated histidine kinase LOV-HK in its illuminated state
Source: Biochem Biophys Rep. 2018 Sep 26;16:39–43. doi: 10.1016/j.bbrep.2018.09.005 (PMC6171537; doi:10.1016/j.bbrep.2018.09.005)
Supplement: Supplementary file 2 — Supplementary material [file mmc2.docx]

**Supplementary Material**


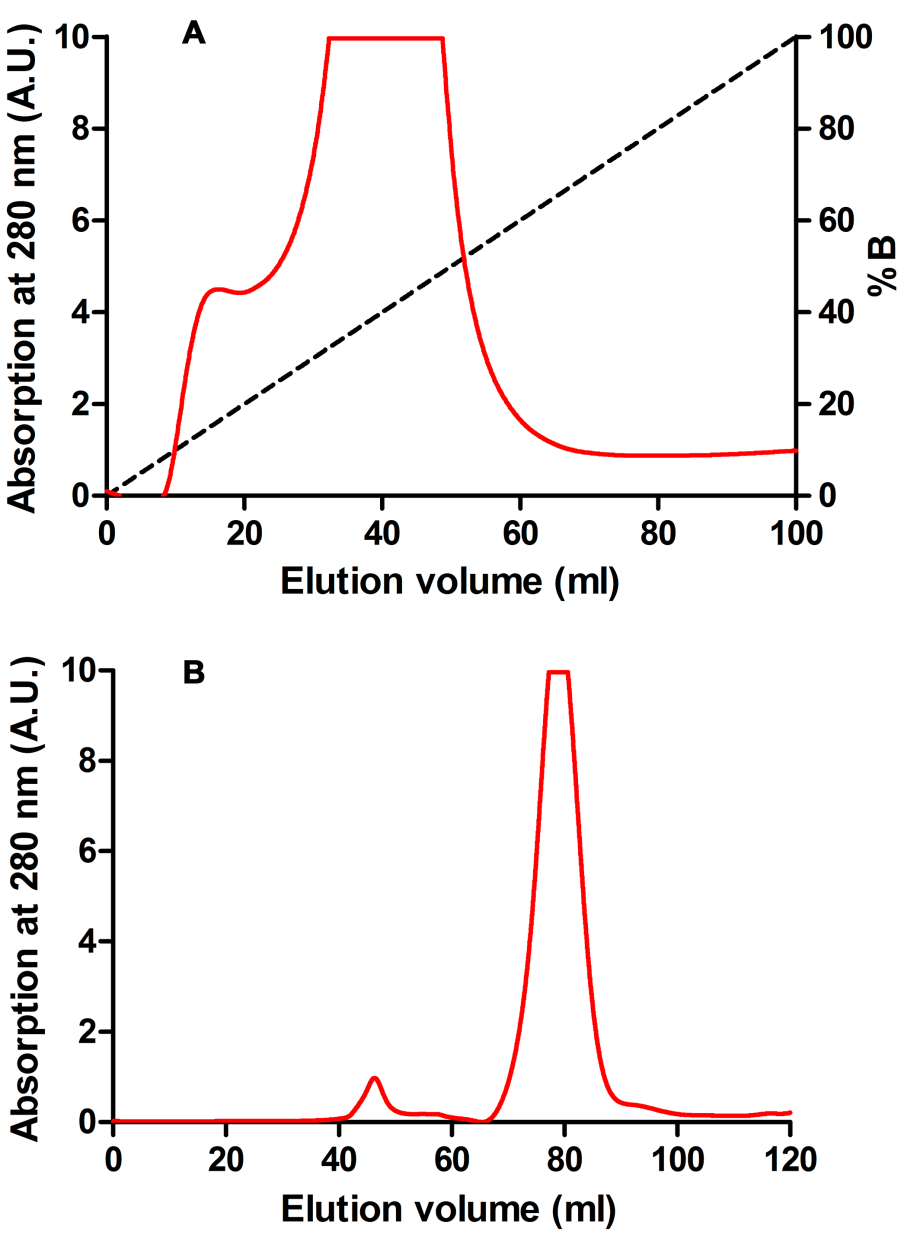


**Supplementary Figure S1.** Chromatographic runs. (A) Nickel-NTA affinity (His-trap HP column). (B) Gel filtration (Superdex 200 16/60 column). The truncated shape in both major peaks is due to saturation of the UV detector in the FPLC machine.


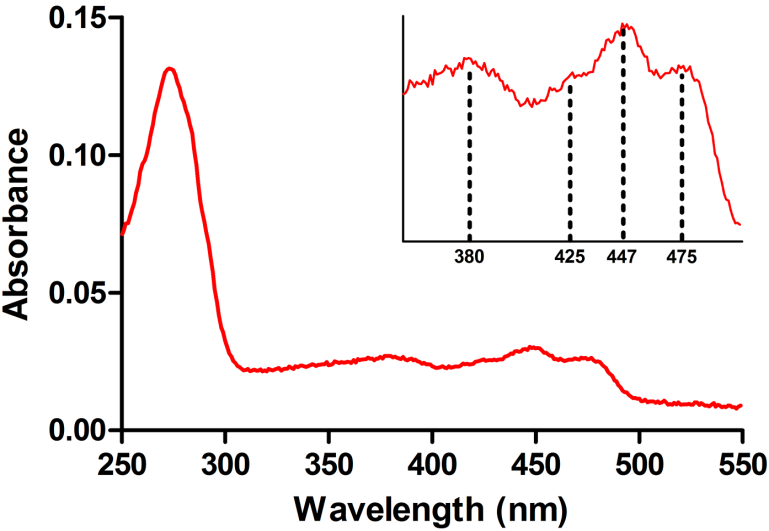


**Supplementary Figure S2.** UV-Vis spectrum of purified LOV-HK (dark state, approximately 1.5 mg ml^-1^). The inset highlights the characteristic absorption peaks of the bound FMN ligand in the dark [3], which serves as a good control for the absence of light contamination during the purification process.


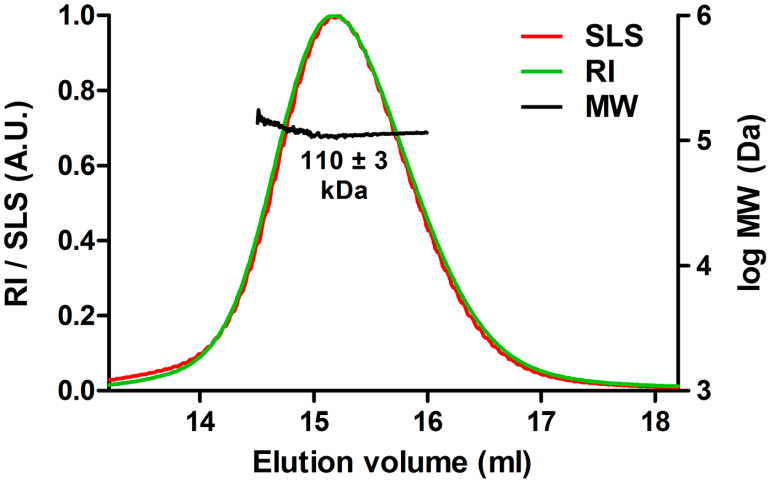


**Supplementary Figure S3.** Determination of the MW of LOV-HK (dark state) by static light scattering (SLS), on a Superdex 200 10/300 column (0.3 µg). SLS at 90º as well as refractive index (RI) signals were monitored. Bovine serum albumin (MW 66.5 kDa) was used as a standard.

**Supplementary Table S1.** Primers and protein sequence information

| Forward primer (cloning artifact M) | gtttaactttaagaaggagatatacatatggcgctatcgcaggcaaccgatccctttcgg |
| --- | --- |
| Reverse primer (cloning artifact HHHHHH) | agccggatctcagtggtggtggtggtggtgcgcgatgcgctgttccggcacattgtggcc |
| Amino acid sequence of the construct produced | MALSQATDPFRAAVEFTLMPMLITNPHLPDNPIVFANPAFLKLTGYEADEVMGRNCRFLQGHGTDPAHVRAIKSAIAAEKPIDIDIINYKKSGEAFWNRLHISPVHNANGRLQHFVSSQLDVTLELSRLVELEKERKTLSIETARSKDQLDYIVEVANIGFWTREFYSGKMTCSAECRRIYGFTPDEPVHFDTILDLVVLEDRMTVVQKAHQAVTGEPYSIEYRIVTRLGETRWLETRAKALTGENPLVLGIVQDVTERKKAEANKALVSREIAHRFKNSMAMVQSIANQTLRNTYDPEQANRLFSERLRALSQAHDMLLKENWAGATIQQICATALAPFNSTFANRIHMSGPHLLVSDRVTVALSLAFYELATNAVKYGALSNEKGVINITWAIMEDKGEKKFHMRWAESRGPEVMQPARRGFGQRLLHSVLAEELKAKCDVEFAASGLLIDVLAPITPEVFPGMGHNVPEQRIAHHHHHH |
